# Immunotherapy with patient-specific antigens selection reduced the metastasis of a cervical cancer patient

**DOI:** 10.1186/2051-1426-3-S2-P385

**Published:** 2015-11-04

**Authors:** Yanyan Han, Jianting Long, Sheng Ye, Ran Tao, Dongyun Wu, Jin Li, Xiangjun Zhou

**Affiliations:** 1 Department of Infectious Diseases, Nanfang Hospital, Southern Medical University, Guangzhou, China; 2 Cell Immunotherapy Center, Dept. of Medicinal Oncology, The First Affiliated Hospital of SUN Yat-Sen University, Guangzhou, China; 3 SYZ Cell Therapy Co., Shenzhen, China

## Background

Cervical cancer is the second most common gynecologic malignant tumor, and is frequently associated with human papilloma virus (HPV) infection. Patients with vascular invaded tumors are more likely to develop metastatic disease after radical resection of primary tumor. Current treatment for metastatic cervical cancer is not effective. In 2011, a patient was diagnosed as HPV^+^ cervical squamous cell carcinoma with vascular invasion. 33 months after radical resection, and subsequent adjuvant chemoradiation therapy, metastasis was detected on the right sacroiliac joint. Given that standard therapy was unsuccessful, the patient was treated with MASCT (Multiple Antigen Stimulating Cellular Therapy), which is composed of multiple tumor antigen pulsed dendritic cells (DCs) and autologous T lymphocytes activated by these DCs.

## Methods

Monocytes from the patient's PBMCs were differentiated into immature dendritic cells (iDCs) and then pulsed with multiple synthetic peptide antigens including tumor-associated antigens and HPV specific antigens. The semi-mature DCs were further stimulated by diverse TLR ligands to differentiate into mature DCs (mDCs). Half of these mDCs were subcutaneous injected to the patient, and the other half were co-cultured with the maintaining non-adherent T cells for another 7-9 days before infusion back to the patient.

## Results

**Figure 1:**
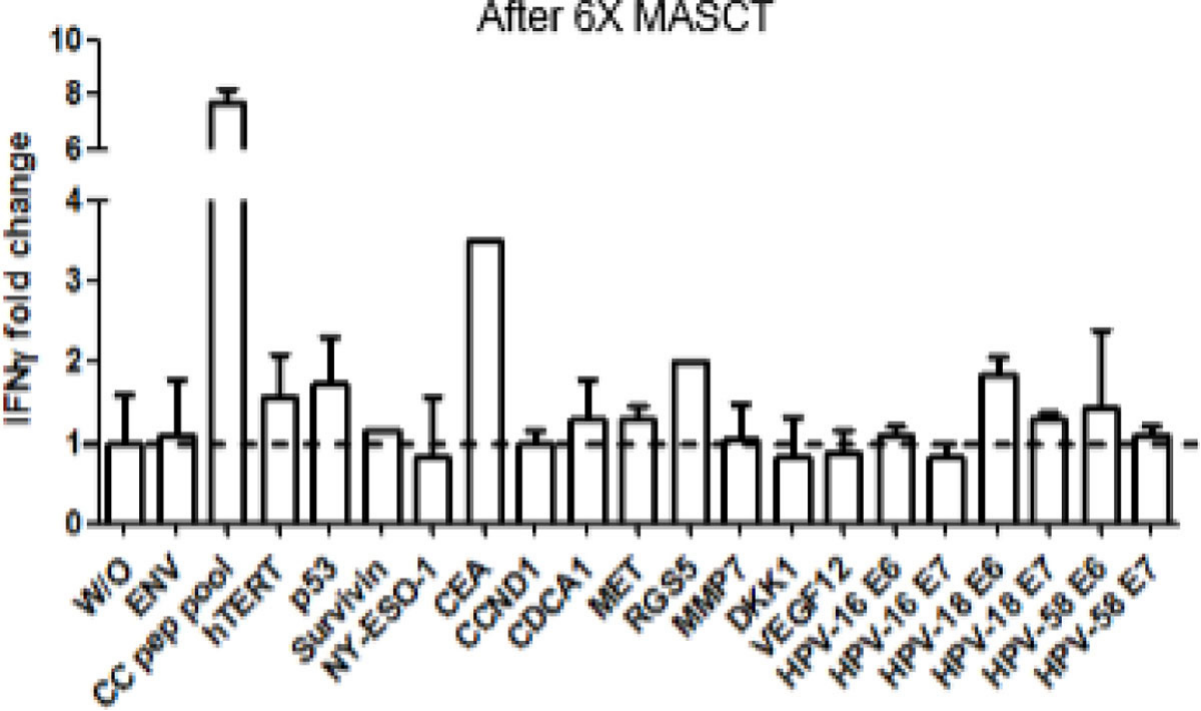


**Figure 2:**
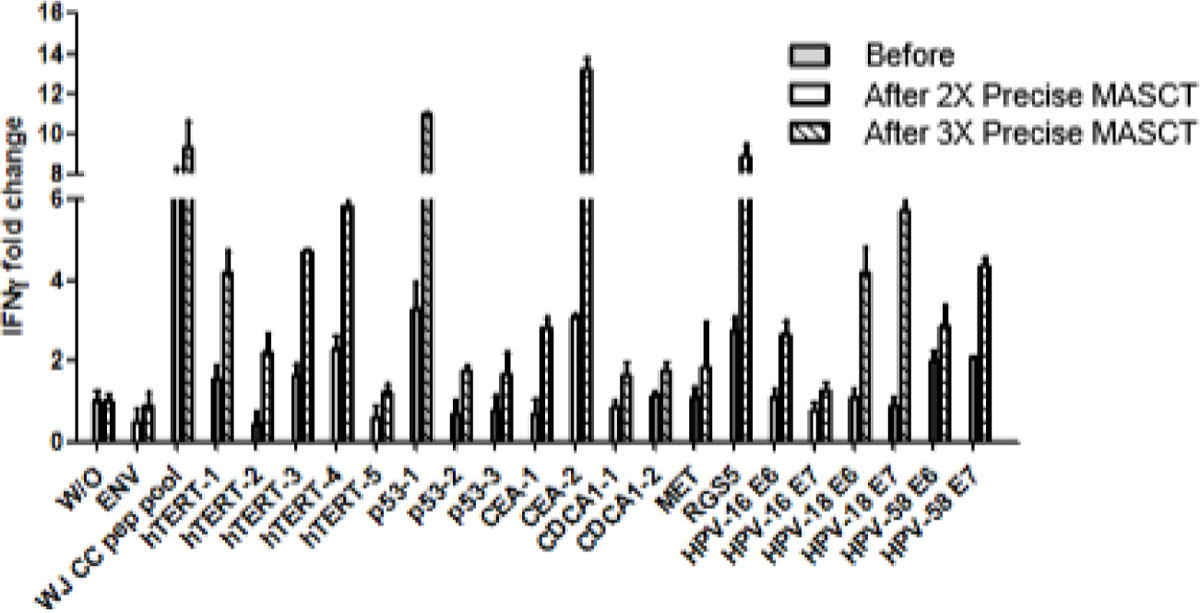


The mDCs expressed high level of HLA-DR, CD80, CD86, CD83, and CCR7 on the surface. And the activated T cells were almost exclusively CD3^+^ T cells, with a major part being CD3^+^CD8^+^ cells and insignificant amounts of regulatory T cells. After local irradiation, the patient was given repeating MASCT treatments every 1-2 months. After 3 times MASCT treatments (6 months after metastasis), the patient was evaluated as partial remission by whole body bone SPECT (single-photon emission computed tomography). Following additional 5 times MASCT treatments (12 months after metastasis), the patient remained partial remission. Moreover, specific responses against tumor antigens were detected in the patients' PBMCs by IFNg ELISPOT assay, such as telomerase, p53, CEA, and HPV18/58 (Figure 1). Based on patient's specific immune response, we therefore adjusted her antigen peptide pool by saving the antigens, which had induced specific responses and removed the peptides that did not. The adjusted antigen pool clearly further boosted the specific responses (Figure 2).

## Conclusion

Our study provides MASCT with as a safe treatment, which has reduced the metastasis of the cervical cancer patient. Moreover, tumor antigens specific T cell responses could be robustly raised in cervical cancer patients after MASCT treatment, and were even further boosted after patient-specific antigens selection.

